# Gastrointestinal Toxicity and Clinical Predictors of Total Neoadjuvant Therapy in Rectal Cancer: A Real-World Retrospective Study

**DOI:** 10.3390/life16030422

**Published:** 2026-03-04

**Authors:** Lucian Dragoș Bratu, Puiu Olivian Stovicek, Ana-Maria Ciurea, Alina Maria Mehedințeanu, Tradian Ciprian Berisha, Ramona Adriana Schenker, Andrei Mircea Dicianu, Carmen Stamulescu, Ștefan Paitici, Stelian Ștefăniță Mogoantă, Michael Schenker

**Affiliations:** 1 Doctoral School, University of Medicine and Pharmacy of Craiova, 200349 Craiova, Romania; lucian.bratu@gmail.com (L.D.B.); berishatc.sfn@gmail.com (T.C.B.); 2 Sf. Nectarie Oncology Center, 200347 Craiova, Romania; amciurea14@gmail.com (A.-M.C.); alina.maria591@gmail.com (A.M.M.); ramona_schenker@yahoo.com (R.A.S.); dicianuandrei@yahoo.com (A.M.D.); carmen_stamulescu@yahoo.com (C.S.); michael.schenker@umfcv.ro (M.S.); 3 Department of Pharmacology, Faculty of Nursing, Târgu Jiu Subsidiary, Titu Maiorescu University, 040441 Bucharest, Romania; 4 Department of Oncology, University of Medicine and Pharmacy of Craiova, 200349 Craiova, Romania; 5 Department of Surgery, University of Medicine and Pharmacy of Craiova, 200349 Craiova, Romania; stefan.paitici@umfcv.ro; 6 3rd General Surgery Clinic, Emergency County Hospital, 200642 Craiova, Romania

**Keywords:** rectal cancer, total neoadjuvant therapy, chemoradiotherapy, gastrointestinal adverse events, treatment-related toxicity

## Abstract

**Background:** Total neoadjuvant therapy (TNT) is increasingly administered in rectal cancer, but compared with concurrent chemoradiotherapy (CRT), data regarding the gastrointestinal (GI) toxicity profile and clinical predictors remain limited. **Objectives:** To evaluate GI toxicity associated with TNT compared with CRT and to explore clinical predictors of these adverse events (AEs). **Methods:** This retrospective study included 201 patients with rectal cancer treated with TNT (*n* = 157) and CRT (*n* = 44). GI AEs (nausea, vomiting, diarrhea) were graded according to CTCAE v5.0. In the analysis of factors associated with GI AEs, multiple clinical and pathological variables were included using multivariable logistic regression. **Results:** The composite endpoint “any GI AEs grade ≥ 1” was more frequent in the TNT group compared with the CRT group (33.1% vs. 15.9%; RR = 2.08; 95% CI 1.02–4.25; *p* = 0.038). Nausea was significantly more frequent in the TNT group (28.7% vs. 9.1%; RR = 3.15; 95% CI 1.20–8.30; *p* = 0.012), whereas vomiting (9.6% vs. 2.3%; *p* = 0.203) and diarrhea (17.8% vs. 9.1%; *p* = 0.242) did not reach statistical significance. In multivariable logistic regression, TNT (OR = 2.65; 95% CI 1.08–6.53; *p* = 0.032) and female sex (OR = 2.03; 95% CI 1.05–3.77; *p* = 0.033) were identified as independent predictors of grade ≥ 1 GI AEs. For nausea, TNT remained significant (OR = 4.37; 95% CI 1.45–13.20; *p* = 0.0089). Upper rectal tumor location was significantly associated with vomiting (*p* = 0.0054). No grade 3–4 GI AEs were observed in either treatment group. **Conclusions:** TNT was associated with a higher incidence of mild GI AEs, predominantly driven by nausea, without an increase in severe toxicities. TNT and female sex were identified as independent clinical predictors of an increased risk of GI AEs, while tumor location in the upper third of the rectum was associated with a higher occurrence of vomiting.

## 1. Introduction

Colorectal cancer remains a major oncological disease, with a significant impact on healthcare systems due to its high incidence and the substantial burden associated with cancer-related morbidity and mortality [1,2]. Among these malignancies, rectal cancer represents a distinct clinical subgroup, accounting for approximately 30–35% of all colorectal cancer cases, and is characterized by a complex therapeutic approach requiring the integration of multimodal treatments to optimize both local and systemic disease control [3]. The anatomical particularities of the rectum, together with its close relationship to adjacent pelvic structures, require carefully sequenced treatment strategies, in which radiotherapy and neoadjuvant chemotherapy play a central role [4,5]. In this context, treatment evaluation extends beyond traditional oncological outcomes to include the assessment of treatment-related toxicity profiles [6]. Furthermore, recent epidemiological trends indicate a shift in the age distribution of patients diagnosed with rectal cancer, with an estimated annual increase of 1–3% in incidence among adults younger than 50 years, underscoring the need for a better understanding of treatment tolerability and short to medium-term quality of life outcomes [7,8].

Radiotherapy constitutes an integral part of rectal cancer management and is used with both curative and palliative intent, depending on disease stage and clinical objectives. Technological advances over recent decades have strengthened the role of radiotherapy through optimization of dose distribution and adaptation to the anatomical and clinical characteristics of each disease stage. The introduction of advanced irradiation techniques, such as intensity-modulated radiotherapy (IMRT) and volumetric modulated arc therapy (VMAT), has improved dose conformity to target volumes and reduced irradiation of adjacent pelvic structures, thereby contributing to a reduction in gastrointestinal (GI) toxicities associated with treatment [3,4,6,9,10].

Rectal cancer treatment relies on a multimodal approach integrating surgery, systemic therapy, and radiotherapy in different sequences and combinations, depending on tumor stage and individual patient characteristics. The selection of the therapeutic strategy and treatment sequencing is determined within a multidisciplinary team, with the aim of maximizing oncological control while minimizing the risk of treatment-related adverse events (AEs).

An important role of radiotherapy in rectal cancer is its integration with chemotherapy in the neoadjuvant setting for patients with locally advanced disease, including T3–T4 tumors and/or regional lymph node involvement (N+) [4]. In the absence of contraindications, radiotherapy is combined with chemotherapy either as concurrent chemoradiotherapy (CRT) or within the more comprehensive strategy of total neoadjuvant therapy (TNT).

The efficacy of neoadjuvant treatment is primarily assessed by postoperative pathological staging, tumor regression grade, particularly by the achievement of a pathological complete response, which is recognized as an important prognostic factor [11]. In recent years, TNT has increasingly been adopted as the preferred therapeutic strategy compared with CRT, owing to superior outcomes reported in terms of disease control and oncological outcomes [5,12,13].

Within the TNT framework, systemic therapy may be administered either as induction chemotherapy followed by CRT or as consolidation chemotherapy following CRT, most commonly using oxaliplatin-based regimens such as CAPEOX (capecitabine and oxaliplatin) or FOLFOX (5-fluorouracil, oxaliplatin, and leucovorin). Radiotherapy is administered concurrently with capecitabine or 5-fluorouracil.

GI AEs associated with radiotherapy in rectal cancer are related to radiation-induced inflammatory processes affecting the small bowel and rectum, clinically manifesting as radiation enteritis and radiation proctitis [6,14,15]. Radiation enteritis is frequently associated with nausea, vomiting, abdominal cramping, and diarrhea, whereas radiation proctitis is characterized by tenesmus, fecal urgency, and rectal bleeding [6,14].

The primary mechanism of radiotherapy-induced GI toxicity involves damage to the intestinal epithelium, leading to impairment of absorptive function, disruption of the mucosal barrier, and inflammatory activation, clinically manifesting as diarrhea, nausea, and vomiting [6,14,15,16].

The incidence and severity of GI AEs vary according to individual anatomical characteristics, the volume of irradiated bowel, administered radiation doses, and the irradiation technique used [14,15,16,17]. This highlights the need for systematic evaluation of GI AEs in the context of modern neoadjuvant strategies.

GI AEs are common during neoadjuvant treatment of rectal cancer, particularly when radiotherapy is combined with chemotherapy. The incidence is typically reported to range between 20% and 40%, with diarrhea, nausea, and vomiting being the most frequent symptoms [18,19,20]. Diarrhea represents the most common toxicity, with reported rates generally between 10 and 25%, whereas nausea and vomiting occur less frequently, usually in fewer than 10–15% of patients, depending on the intensity of systemic treatment [4,18,19,20].

Dose distribution at the intestinal level is an important determinant of GI toxicity, particularly for diarrhea. Exposure of intestinal volumes to doses exceeding 15 Gy has been associated with an increased risk of GI AEs [21,22,23,24]. Modern radiotherapy techniques have reduced bowel irradiation compared with conventional approaches, contributing to improved treatment tolerability [25,26,27]. These findings support the dose–volume relationship as a central mechanism underlying GI toxicity induced by pelvic radiotherapy [28,29,30].

Despite technological advances and optimization of therapeutic regimens, data on GI AEs associated with neoadjuvant treatment of rectal cancer remain heterogeneous and incompletely characterized. Most studies primarily focus on oncological outcomes, while comparative data between TNT and CRT and the role of individual clinical predictors of GI toxicity remain limited.

In this context, a more detailed characterization of GI toxicities may optimize therapeutic planning and help identify patients who could benefit from early supportive measures, including guideline-based prophylaxis for radiotherapy- and chemotherapy-induced nausea, vomiting [31,32], and diarrhea [33]. These data may also inform multidisciplinary supportive interventions aimed at maintaining treatment tolerability and continuity.

The aim of this study was to evaluate the incidence and pattern of GI toxicities and to explore clinical predictors of these events in patients with rectal cancer treated with neoadjuvant therapy, with a focus on nausea, vomiting, and diarrhea. Specifically, we aimed to compare the GI toxicity profile of TNT with CRT and to analyze the association between the occurrence of GI AEs and a selected set of clinical and therapeutic factors. In clinical practice, the results may be useful in identifying patients at higher risk of GI toxicity who may benefit from closer clinical monitoring and the implementation of tailored supportive measures.

## 2. Materials and Methods

In this retrospective study, we analyzed the incidence of GI AEs associated with neoadjuvant radiotherapy in the treatment of rectal cancer. A total of 201 patients treated at Sf. Nectarie Oncology Center, Craiova, between 2020 and 2023, was included. Inclusion criteria were: age ≥ 18 years; histopathologically confirmed rectal adenocarcinoma; indication for neoadjuvant treatment established by a multidisciplinary oncology board; treatment with either total neoadjuvant therapy (TNT) or concurrent chemoradiotherapy (CRT); and complete documentation regarding the occurrence and severity of GI AEs during neoadjuvant treatment. Exclusion criteria were: incomplete toxicity documentation and absence of post-therapeutic evaluation. Of the 201 patients, 157 received TNT with either FOLFOX or CAPEOX regimens, while 44 patients underwent CRT with capecitabine. Clinical, pathological, and therapeutic data were extracted from medical records and institutional databases. GI AEs were recorded throughout the course of neoadjuvant treatment.

The inclusion and exclusion criteria were defined to ensure the homogeneity of the analyzed cohorts and to allow a comparable evaluation of GI toxicities associated with standard neoadjuvant treatment. Only patients treated with conventional CRT or TNT regimens were included, in order to avoid heterogeneity related to alternative protocols (e.g., short-course radiotherapy). Exclusion of patients who did not complete treatment or who lacked post-therapeutic imaging evaluation was necessary to allow appropriate analysis of postoperative staging and stage modification, variables subsequently incorporated into the statistical models.

In the analyzed cohort, no surgical interventions or diverting procedures were performed as a result of acute GI complications occurring during neoadjuvant treatment.

The indication for neoadjuvant treatment was established by the multidisciplinary oncology board based on clinical staging and tumor characteristics, in accordance with international guidelines and institutional protocols. Neoadjuvant treatment was primarily recommended for patients with locally advanced rectal tumors, defined by T3–T4 tumor invasion and/or the presence of regional lymph node involvement, as well as in selected cases of oligometastatic disease amenable to surgical resection. Patients were treated either with CRT or with TNT, according to the institutional protocols in use at the time of treatment. Standard chemoradiotherapy consisted of conventionally fractionated long-course radiotherapy administered concurrently with oral capecitabine. Total neoadjuvant therapy included the administration of oxaliplatin-based systemic chemotherapy prior to chemoradiotherapy, using standard regimens, followed by conventionally fractionated radiotherapy. The choice of therapeutic regimen was made on an individual basis within the multidisciplinary team, according to the patient’s clinical status and the overall therapeutic strategy.

A dedicated dosimetric–volumetric analysis was not performed, as the primary objective of the study was to evaluate the clinical incidence and predictors of GI toxicities, and detailed dosimetric parameters were not uniformly available for all patients due to the retrospective design.

GI AEs were monitored throughout the course of neoadjuvant treatment and were classified according to the Common Terminology Criteria for Adverse Events (CTCAE v5.0), thus ensuring standardized grading of symptom severity [34]. GI AEs were evaluated and documented during the course of treatment in accordance with routine clinical practice, and the data were subsequently retrospectively extracted from medical records for statistical analysis.

Management of GI AEs was performed using symptomatic treatment, in accordance with current clinical practice and international guideline recommendations. Nausea and vomiting were treated with hydration and the administration of antiemetic medication, including metoclopramide or granisetron, depending on symptom severity and clinical response. Diarrhea was managed with the administration of loperamide, in association with supportive measures such as dietary adjustments, probiotic treatment, and oral or intravenous hydration, as clinically indicated. The choice and intensity of symptomatic treatment were individually adapted according to the grade of the AEs and the patient’s clinical status.

The analysis focused on the incidence of GI AEs (nausea, vomiting, and diarrhea), and a composite endpoint was defined, representing the occurrence of any GI AEs of grade ≥ 1 (ANY_GE1) during neoadjuvant treatment. For the analysis of factors associated with GI AEs, relevant clinical and pathological variables were considered, including patient age and sex, tumor differentiation grade, initial tumor stage, rectal tumor location, postoperative stage, stage modification after neoadjuvant treatment, and the type of neoadjuvant treatment administered.

The study was conducted in accordance with the principles of the Declaration of Helsinki. All included patients provided written informed consent for the use of clinical and therapeutic data for research purposes. The study protocol and the use of medical data were approved by the institutional ethics committee, in accordance with applicable local and national regulations.

### Statistical Analysis

Statistical analyses were performed on a complete dataset, as patients with incomplete clinical or toxicity documentation were excluded at the eligibility stage, prior to analysis.

Descriptive methods, univariate and multivariable inferential tests, as well as advanced adjustment models and exploratory analyses of relationships between variables, were applied. Categorical variables were presented as frequencies and percentages, while continuous variables were expressed as mean ± standard deviation. Comparisons of characteristics between therapeutic groups (TNT vs. CRT) and across categories of clinical variables were performed using the Chi-square test or Fisher’s exact test, depending on sample size and frequency distribution. To assess differences in the distribution of AEs grades (grade 0–grade 2) across ordinal variables (age: <50, 50–69, ≥70 years; tumor grade: G1-G3; postoperative stage), Cochran–Armitage trend tests were applied to identify potential linear trends in the risk of AEs. For multicategorical variables that showed a significant global effect (e.g., rectal tumor location: upper, middle, lower), the analysis was complemented by pairwise post hoc comparisons using Fisher’s exact test to identify specific differences between categories.

To estimate the probability of GI AEs, binary logistic regression models were constructed, including relevant clinical variables (age, sex, rectal tumor location, and treatment type). The maximum severity of GI AEs (grade 0–grade 2) was evaluated using an ordinal cumulative logit regression model (proportional odds model), which is appropriate for modeling incremental severity of AEs. Model validity was assessed by examining coefficient significance, confidence intervals, and the proportional odds assumption. Multicollinearity among the predictors included in the multivariable models was assessed by calculating variance inflation factors (VIF). All VIF values were below the threshold of 5 (range 1.43–4.87), supporting the stability of the estimated effects.

Given the marked imbalance in sample size between the TNT and CRT groups, a propensity score–based method using inverse probability of treatment weighting (IPTW) was applied to adjust for baseline differences between treatment groups. Propensity scores were estimated using logistic regression, including baseline clinical variables (age, sex, tumor location, tumor grade, and initial stage). The weighted model was subsequently used in a logistic regression analysis to estimate the adjusted treatment effect on the binary composite endpoints: occurrence of ANY_GE1 and occurrence of grade 2 GI AEs.

The co-occurrence of different types of GI AEs (nausea, vomiting, and diarrhea) was assessed using the phi (φ) coefficient, which quantifies the association between two binary variables. To synthesize estimates obtained using different statistical approaches (logistic regression, ordinal regression, and IPTW), a forest plot was constructed to graphically display odds ratios (ORs) and 95% confidence intervals, allowing comparison of the effects of TNT and female sex on the risk of GI AEs. All statistical tests were two-tailed, and *p* values < 0.05 were considered statistically significant.

## 3. Results

The study cohort included 116 male (57.7%) and 85 female (42.3%) patients, with the majority aged between 50 and 69 years (*n* = 106).

The distribution of GI AEs according to demographic and clinicopathological characteristics is presented in Table 1. Nausea was the most frequent GI AE of grade ≥ 1, with incidences ranging between 20.7% and 29.4% according to sex and between 20.7% and 35.9% according to rectal tumor location, with the highest values observed in stage IVA (60.0%). Diarrhea was less frequent in most subgroups (13.6–18.5%) but increased substantially in advanced stages, particularly stage IVA (40.0%). Vomiting of grade ≥ 1 was rare, with incidences generally below 10%, reaching higher values only in subgroups with a small number of patients. Grade 2 GI AEs were infrequent across all analyzed categories, and no grade 3 or 4 events were reported.

Table 2 allows the correlation of postoperative stage and type of tumor evolution (downstaging, complete response, stable disease, or upstaging) with the distribution of GI AEs, without demonstrating a consistent pattern of AEs according to stage evolution.

Grade ≥ 1 nausea ranged from 23.7% in postoperative stage I to 36.4% in stage IIIB, without a clear progressive pattern. Grade ≥ 1 diarrhea was observed in 16.3% of patients with stage I and 24.3% in stage IIA. Grade ≥ 1 vomiting was generally below 10% across most categories. According to stage modification, grade ≥ 1 nausea was reported in 23.8% of patients with downstaging, 24.3% in those with complete response, and 23.5% in stable disease, while grade ≥ 1 diarrhea ranged between 13.5% and 18.2% in these groups. Grade 2 events were rare, and no grade 3 or 4 events were reported.

Advanced statistical analyses were subsequently performed to evaluate treatment-related differences and identify clinical predictors of GI AEs. Comparative analyses between treatment groups are presented in Table 3, univariate and trend analyses in Table 4, multivariable regression models in Table 5 and Table 6, and sensitivity analyses in Table 7.

The incidence and grade distribution of GI AEs according to treatment regimen (TNT vs. CRT), together with risk ratios (RRs), are presented in Table 3. Grade ≥ 1 nausea was significantly more frequent in the TNT arm compared with CRT (28.7% vs. 9.1%; RR = 3.15, 95% CI 1.20–8.30; *p* = 0.012), indicating a 3.15-fold higher risk associated with TNT. For grade ≥ 1 vomiting, the incidence was higher in the TNT arm (9.6%) compared with CRT (2.3%); however, the difference did not reach statistical significance (RR = 4.20, 95% CI 0.57–30.95; *p* = 0.203), possibly due to the small number of events. Grade ≥ 1 diarrhea also showed a higher frequency in the TNT group (17.8% vs. 9.1%), without statistical significance (RR = 1.96, 95% CI 0.73–5.29; *p* = 0.242). The composite endpoint ANY_GE1 was significantly more frequent in the TNT arm (33.1% vs. 15.9%; RR = 2.08, 95% CI 1.02–4.25; *p* = 0.038), corresponding to an approximately two-fold higher probability of GI AEs in the TNT arm. Grade 2 GI AEs were rare in both groups and did not show statistically significant differences between treatments.

Univariate and trend analyses of clinical factors associated with grade ≥ 1 GI AEs are presented in Table 4. Univariate analysis did not identify significant associations between age, sex, tumor differentiation grade, initial tumor stage, postoperative stage, or stage modification and the occurrence of grade ≥ 1 nausea or diarrhea (all *p* values > 0.05). Values close to the threshold of statistical significance were observed for the postoperative stage in the case of nausea (*p* = 0.099) and for the initial stage in the case of diarrhea (*p* = 0.101), without reaching statistical significance. Rectal tumor location was the only clinical variable significantly associated with the occurrence of grade ≥ 1 vomiting in the global analysis (*p* = 0.0054), with patients presenting tumors located in the upper third of the rectum showing a higher frequency compared with middle and lower locations. For nausea and diarrhea, tumor location did not show significant differences. Trend analysis applied to the included ordinal variables (age, tumor grade, and postoperative stage) did not identify significant linear relationships between category order and the incidence of grade ≥ 1 GI AEs. Rates of nausea, vomiting, and diarrhea varied across subgroups without a consistent gradient, and trend tests were non-significant for all types of AEs analyzed (all *p* values > 0.05).

The results of the multivariable analyses (logistic and ordinal) are presented in Table 5. In the logistic regression model, with the occurrence of ANY_GE1 as the endpoint, female sex and TNT treatment remained significant independent predictors. Female sex was independently associated with an approximately twofold increased risk of GI AEs (OR = 2.03; 95% CI 1.05–3.77; *p* = 0.033), while TNT treatment was associated with an approximately 2.6-fold increase in the odds of developing at least one grade ≥ 1 GI AE compared with CRT (OR = 2.65; 95% CI 1.08–6.53; *p* = 0.032). Tumor location in the upper third of the rectum showed a borderline association with the risk of GI AEs (OR = 2.09 vs. lower rectum; *p* = 0.081), without reaching statistical significance, whereas age did not significantly influence the probability of GI AEs (OR = 1.01 per year; *p* = 0.50). The results of the ordinal regression were concordant with those of the logistic model, both in terms of effect direction and magnitude of the estimates. Female sex (OR = 1.92; 95% CI 1.03–3.59; *p* = 0.04) and TNT treatment (OR = 2.62; 95% CI 1.04–6.60; *p* = 0.038) were associated with an increased probability of belonging to a higher severity level. Upper rectal tumor location again showed a borderline association (OR = 2.12; *p* = 0.075), whereas age and middle rectal tumor location did not have a significant independent contribution.

Multivariable logistic regression models for GI AEs ≥ grade 1, analyzed separately and as a composite endpoint, are presented in Table 6. For the composite endpoint ANY_GE1, TNT treatment and female sex remained independently associated with the risk of GI AEs. TNT treatment was associated with a significant increase in risk (OR = 2.84; 95% CI 1.16–6.97; *p* = 0.0227), while female sex was associated with an approximately twofold increased risk (OR = 1.91; 95% CI 1.02–3.58; *p* = 0.043). Age did not have a significant effect on this endpoint (*p* = 0.6454).

In the separate analysis by type of GI AEs, only grade ≥ 1 nausea was significantly associated with TNT treatment. Patients treated with TNT had a more than fourfold higher risk of grade ≥ 1 nausea compared with those treated with CRT (OR = 4.37; 95% CI 1.45–13.20; *p* = 0.0089). Female sex and age were not significantly associated with the risk of nausea. For grade ≥ 1 vomiting and grade ≥ 1 diarrhea, none of the predictors included in the models (age, sex, and treatment type) showed statistically significant associations (all *p* values > 0.05). Overall, the difference observed for the composite endpoint ANY_GE1 is mainly driven by the increased incidence of grade ≥ 1 nausea in the TNT arm, while vomiting and diarrhea had limited and non-significant contributions to the overall differences in GI AEs.

Sensitivity analyses were performed to assess the consistency of the main results regarding GI AEs, as shown in Table 7. Evaluation of grade 2 AEs according to clinical characteristics did not reveal significant associations for nausea or vomiting (all *p* values > 0.25). In contrast, grade 2 diarrhea was significantly associated with initial tumor stage (*p* = 0.0143), with more severe episodes predominantly observed in advanced stages, suggesting a possible impact of disease extent on diarrhea severity.

Repeating the multivariable logistic analysis for the endpoint AE ≥ grade 1 confirmed the stability of the predictors identified in the main models. TNT treatment and female sex remained associated with an increased risk of GI AEs, with *p* values within the range of statistical significance or borderline significance, while age did not demonstrate an independent effect.

Analysis of grade 2 AEs according to treatment type showed a low number of events, particularly in the CRT arm. Although the odds ratio suggested a higher risk in the TNT group (OR = 2.72), the difference did not reach statistical significance (*p* = 0.256), limiting the ability to draw firm conclusions regarding AEs of this grade.

Subgroup analysis for patients aged between 50 and 80 years confirmed the consistency of the main results. TNT treatment remained associated with the occurrence of ANY_GE1, with a borderline result (*p* = 0.050), while female sex and upper rectal tumor location showed statistically non-significant trends. Overall, these sensitivity analyses support the direction of the associations observed in the main analyses, indicating that TNT treatment remains associated with more GI AEs in comparison to CRT, and that influence of female gender is maintained in the same direction across sensitivity analyses.

**Figure 1:**
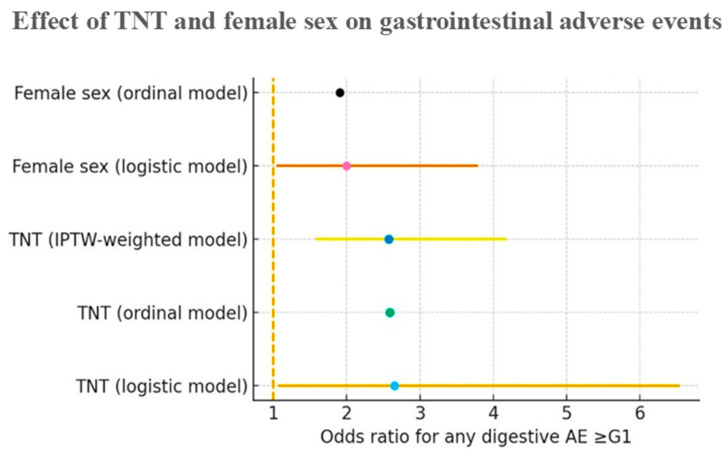
Forest plot of the effect of TNT treatment and female sex on the risk of GI AEs ≥ grade 1 based on logistic regression, ordinal regression, and IPTW analysis. Absolute numbers and corresponding percentages for the composite endpoint ANY_GE1 were 52/157 (33.1%) in the TNT group and 7/44 (15.9%) in the CRT group. Dots represent odds ratios (OR), and horizontal lines indicate 95% confidence intervals (CI). The dashed vertical line represents OR = 1 (no effect).

The estimates of the independent effect of TNT treatment and female sex on the risk of grade ≥ 1 GI AEs were evaluated using three complementary statistical models: binary logistic regression, ordinal regression, and inverse probability of treatment weighting (IPTW) analysis (Figure 1). Estimates derived from the ordinal regression models are presented to compare the direction of associations and are not directly comparable with the risk estimates obtained from binary logistic models. For the logistic regression and IPTW models, odds ratios (ORs) with 95% confidence intervals are shown. For the ordinal model, exponentiated coefficients are displayed to indicate the direction and relative magnitude of the association. The vertical dashed line indicates the neutrality value (OR = 1). Across all applied models, TNT treatment was associated with an increased risk of ANY_GE1 (ORs 2.57–2.65), and female sex showed a consistent association with a higher risk of GI AEs (ORs 1.90–2.03). Confidence intervals are not reported for the ordinal model because the estimates are derived from proportional log-odds coefficients and are not directly comparable with those from binary logistic models. The convergence of OR values across methods indicates consistency of the results and stability of the effect of the estimates.

To assess the consistency of the observed results and to explore additional relationships between GI AEs and clinical characteristics, post hoc analyses and additional multivariable models were performed. Post hoc analyses revealed a significant difference in the distribution of grade ≥ 1 vomiting according to rectal tumor location: tumors located in the upper rectal tumor location showed significantly higher rates compared with middle and lower locations (upper vs. lower: *p* = 0.0212; upper vs. middle: *p* = 0.0083). Ordinal variables (age, grade, postoperative stage) did not show significant linear trends. Additional multivariable models confirmed the independent association of female sex and TNT treatment with the severity of GI AEs, while age and tumor location did not demonstrate statistically significant effects. The results were confirmed by standard logistic regression (OR for TNT = 2.65; 95% CI 1.08–6.53) and by IPTW analysis (OR = 2.57; 95% CI 1.59–4.17; *p* = 0.00012). Correlation analysis showed moderate associations between nausea and vomiting (φ = 0.454) and between nausea and diarrhea (φ = 0.466), whereas vomiting and diarrhea showed minimal correlation (φ = 0.053).

## 4. Discussion

The present results indicate that total neoadjuvant therapy (TNT) is associated with a higher incidence of GI AEs compared with concurrent chemoradiotherapy (CRT), predominantly driven by an increased frequency of mild symptoms, without a proportional rise in severe toxicities. This pattern suggests that TNT primarily influences short-term treatment tolerability rather than escalating overall GI toxicity severity [4,12]. Similar trends have been reported in randomized trials such as RAPIDO and PRODIGE 23, where TNT was associated with a higher rate of mostly low-grade GI AEs without compromising overall safety [5,13]. Although differences in radiotherapy techniques, chemotherapy sequencing, and toxicity reporting limit direct quantitative comparisons across studies [5,13], the present real-world data support the concept of a distinct TNT-related GI toxicity profile. In parallel, evidence indicating improved downstaging and pathological complete response rates with TNT further consolidates its role as a preferred strategy in locally advanced rectal cancer [35]. The reported clinical benefits, favorable tolerability profile, and positive impact on quality of life support the continued interest in this therapeutic approach in clinical practice [36,37].

In our study, nausea emerged as the principal contributor to the increased GI toxicity observed in the TNT group, largely accounting for the difference between TNT and CRT, while vomiting and diarrhea had a limited impact on overall between-group variation. This finding aligns with previous reports indicating that TNT-based regimens are frequently associated with a higher incidence of predominantly low-grade nausea, without consistent increases in severe GI AEs [38,39]. Although typically mild in intensity, nausea may substantially affect quality of life and treatment adherence, highlighting the importance of early recognition and guideline-based supportive management [31,32]. Prospective data using standardized quality-of-life instruments further confirm that even low-grade GI symptoms can significantly impair physical and emotional functioning during pelvic radiotherapy [40]. Recent analyses evaluating toxicity and treatment compliance during total neoadjuvant therapy have reported a predominance of low-grade GI AEs, without a significant increase in grade ≥ 3 toxicity [39,41]. These considerations support systematic symptom monitoring and timely supportive interventions in patients undergoing TNT [31,32,33].

Multivariable analyses identified female sex as an independent risk factor for GI AEs, irrespective of treatment strategy. Similar observations have been reported in studies evaluating pelvic radiotherapy and chemoradiotherapy toxicity profiles [26,42], suggesting increased susceptibility of female patients to GI symptoms. Although the underlying mechanisms remain incompletely defined, proposed explanations include anatomical differences potentially leading to greater small bowel exposure, as well as hormonal and functional factors influencing intestinal motility, mucosal permeability, and inflammatory response [16,17,28]. While these hypotheses are biologically plausible, further prospective research integrating anatomical and dosimetric parameters is needed to clarify this association. Clinically, female sex may therefore represent a relevant factor in risk stratification and supportive monitoring, without implying the need for modification of standard neoadjuvant regimens.

Tumor location in the upper third of the rectum was associated with a higher occurrence of vomiting in both global and post hoc analyses. A possible explanation relates to anatomical and planning considerations, as irradiation of upper rectal tumors may involve larger volumes of small bowel, thereby increasing exposure to clinically relevant doses. The dose–volume relationship at the small bowel level is a recognized determinant of GI toxicity in pelvic radiotherapy [9,21,24], and modern techniques such as IMRT/VMAT have demonstrated reductions in bowel irradiation and GI complications [9,10,42,43,44].

However, in the absence of a dedicated dosimetric–volumetric analysis in the present study, this finding should be interpreted with caution. The observed association may reflect the combined influence of anatomical factors, treatment planning characteristics, and individual variability, and should be considered hypothesis-generating pending confirmation in prospective studies incorporating detailed dosimetric parameters.

Age, tumor grade, initial stage, postoperative stage, and stage modification were not significantly associated with the occurrence of GI AEs, and trend analyses did not demonstrate relationships dependent on disease severity. This pattern suggests that tumor-related clinical characteristics may play a limited role in predicting acute GI toxicity in the neoadjuvant setting. These findings are consistent with evidence indicating that treatment-related characteristics and dosimetric–volumetric factors represent the most robust predictors of pelvic radiotherapy-induced toxicity, whereas disease-related clinical variables are less consistently implicated [45]. Similar observations have been reported in rectal cancer cohorts treated with chemoradiotherapy, where irradiated bowel volumes and treatment planning parameters appear more influential than age or tumor stage in determining GI toxicity risk [42,46].

Collectively, these results reinforce the concept that GI toxicity in the neoadjuvant management of rectal cancer is primarily driven by treatment-related and individual susceptibility factors rather than by tumor burden or oncological stage.

As total neoadjuvant therapy has become increasingly adopted in routine clinical practice, the present study contributes real-world data from a relatively large and heterogeneous cohort treated with TNT or CRT. Future research should aim to clarify the biological and anatomical mechanisms underlying the increased susceptibility observed in female patients and to integrate dosimetric–volumetric parameters into prospective toxicity modeling. Additionally, prospective evaluation of tailored antiemetic and antidiarrheal strategies in the context of TNT may further optimize treatment tolerability without compromising oncological outcomes.

Several limitations inherent to the study design should be acknowledged when interpreting the present findings. The retrospective design implies that GI toxicities were assessed based on existing clinical documentation, without the use of standardized tools for prospective symptom collection. The unequal distribution of patients between the TNT and CRT groups may affect the direct comparability of results, despite the use of multivariable analyses and sensitivity analyses to control for confounding factors. In addition, the analysis focused exclusively on clinically reported GI toxicities, without integrating patient-reported outcome assessments, which could have provided a more detailed characterization of the clinical impact of mild symptoms. The absence of a dedicated dosimetric–volumetric analysis limits the exploration of anatomical and radiotherapy planning mechanisms involved in the development of GI toxicities and in the associations observed with certain clinical characteristics. Nevertheless, the cohort size and the use of complementary statistical methods support the consistency of the results and allow for the formulation of hypotheses that require validation in prospective studies incorporating dosimetric–volumetric parameters and standardized symptom assessments.

## 5. Conclusions

This study highlights that total neoadjuvant therapy (TNT) is associated with a higher incidence of GI AEs compared with concurrent chemoradiotherapy (CRT). Nausea represented the dominant component of this toxicity profile. Multivariable and sensitivity analyses identified TNT and female sex as independent factors associated with an increased risk of GI toxicity, while tumor location in the upper third of the rectum was associated with a higher occurrence of vomiting. Grade 2 GI AEs were infrequent, and no severe (grade 3–4) AEs were observed, suggesting an overall favorable tolerability profile for neoadjuvant treatment strategies. These findings may contribute to improved risk stratification for GI toxicity in patients with rectal cancer treated with neoadjuvant therapy and may support the identification of patients who could benefit from closer clinical monitoring and early implementation of tailored supportive measures.

## Figures and Tables

**Table 1:** Descriptive distribution of gastrointestinal adverse events according to baseline demographic and tumor characteristics.

Variable	Category	*N*	Nausea (Grade) *n* (%)			Vomiting (Grade) *n* (%)			Diarrhea (Grade) *n* (%)		
			0	1	2	0	1	2	0	1	2
Age	<50	22	16 (72.7)	5 (22.7)	1 (4.6)	19 (86.4)	1 (4.5)	2 (9.1)	19 (86.4)	1 (4.5)	2 (9.1)
	50–69	106	80 (75.5)	18 (17.0)	8 (7.5)	97 (91.5)	8 (7.5)	1 (0.9)	87 (82.1)	11 (10.4)	8 (7.5)
	≥70	73	56 (76.7)	15 (20.5)	2 (2.8)	69 (94.5)	3 (4.1)	1 (1.4)	63 (86.3)	8 (11.0)	2 (2.7)
Sex	M	116	92 (79.3)	17 (14.7)	7 (6.0)	106 (91.4)	7 (6.0)	3 (2.6)	99 (85.3)	9 (7.8)	8 (6.9)
	F	85	60 (70.6)	21 (24.7)	4 (4.7)	79 (92.9)	5 (5.9)	1 (1.2)	70 (82.4)	11 (12.9)	4 (4.7)
Tumor location	S	39	25 (64.1)	12 (30.8)	2 (5.1)	36 (92.3)	2 (5.1)	1 (2.6)	32 (82.1)	3 (7.7)	4 (10.2)
	I	75	58 (77.3)	12 (16.0)	5 (6.7)	71 (94.7)	2 (2.7)	2 (2.7)	64 (85.3)	7 (9.3)	4 (5.4)
	M	87	69 (79.3)	14 (16.1)	4 (4.6)	83 (95.4)	3 (3.4)	1 (1.2)	73 (83.9)	10 (11.5)	4 (4.6)
Tumor grade	G1	65	50 (76.9)	11 (16.9)	4 (6.2)	59 (90.8)	4 (6.2)	2 (3.1)	53 (81.5)	9 (13.8)	3 (4.7)
	G2	90	67 (74.4)	19 (21.1)	4 (4.5)	82 (91.1)	6 (6.7)	2 (2.2)	77 (85.6)	6 (6.7)	7 (7.7)
	G3	46	35 (76.1)	8 (17.4)	3 (6.5)	44 (95.7)	2 (4.3)	0 (0.0)	40 (87.0)	2 (4.3)	4 (8.7)
Initial stage	IIA	18	14 (77.8)	1 (5.6)	3 (16.7)	16 (88.9)	1 (5.6)	1 (5.6)	15 (83.3)	3 (16.7)	0 (0.0)
	IIB	5	4 (80.0)	1 (20.0)	0 (0.0)	5 (100)	0 (0.0)	0 (0.0)	5 (100)	0 (0.0)	0 (0.0)
	IIIA	28	22 (78.6)	5 (17.9)	1 (3.6)	25 (89.3)	1 (3.6)	2 (7.1)	26 (92.9)	2 (7.1)	0 (0.0)
	IIIB	108	85 (78.7)	19 (17.6)	4 (3.7)	100 (92.6)	7 (6.5)	1 (0.9)	93 (86.1)	9 (8.3)	6 (5.6)
	IIIC	32	23 (71.9)	7 (21.9)	2 (6.3)	31 (96.9)	1 (3.1)	0 (0.0)	24 (75.0)	5 (15.6)	3 (9.4)
	IVA	10	4 (40.0)	5 (50.0)	1 (10.0)	8 (80.0)	2 (20.0)	0 (0.0)	6 (60.0)	1 (10.0)	3 (30.0)

S = upper rectum; I = middle rectum; M = lower rectum.

**Table 2:** Descriptive distribution of gastrointestinal adverse events according to postoperative stage and stage evolution.

Variable	Category	*N*	Nausea (Grade) *n* (%)			Vomiting (Grade) *n* (%)			Diarrhea (Grade) *n* (%)		
			0	1	2	0	1	2	0	1	2
Post- operative stage	I	80	61 (76.3)	14 (17.5)	5 (6.2)	73 (91.3)	7 (8.7)	0 (0.0)	67 (83.8)	7 (8.8)	6 (7.5)
	IIA	37	25 (67.6)	10 (27.0)	2 (5.4)	34 (91.9)	2 (5.4)	1 (2.7)	28 (75.7)	7 (18.9)	2 (5.4)
	IIB	4	1 (25.0)	2 (50.0)	1 (25.0)	1 (25.0)	2 (50.0)	1 (25.0)	1 (25.0)	1 (25.0)	2 (50.0)
	IIC	1	0 (0.0)	1 (100)	0 (0.0)	0 (0.0)	1 (100)	0 (0.0)	0 (0.0)	0 (0.0)	1 (100)
	IIIA	14	9 (64.3)	4 (28.6)	1 (7.1)	11 (78.6)	3 (21.4)	0 (0.0)	12 (85.7)	1 (7.1)	1 (7.1)
	IIIB	22	14 (63.6)	6 (27.3)	2 (9.1)	18 (81.8)	3 (13.6)	1 (4.5)	19 (86.4)	2 (9.1)	1 (4.5)
	IIIC	6	4 (66.7)	1 (16.7)	1 (16.7)	6 (100)	0 (0.0)	0 (0.0)	4 (66.7)	0 (0.0)	2 (33.3)
	pCR	37	28 (75.7)	6 (16.2)	3 (8.1)	35 (94.6)	1 (2.7)	1 (2.7)	32 (86.5)	3 (8.1)	2 (5.4)
Stage modification	D	143	109 (76.2)	26 (18.2)	8 (5.6)	134 (93.7)	8 (5.6)	1 (0.7)	117 (81.8)	15 (10.5)	11 (7.7)
	C	37	28 (75.7)	7 (18.9)	2 (5.4)	34 (91.9)	1 (2.7)	2 (5.4)	32 (86.5)	5 (13.5)	0 (0.0)
	U	4	2 (50.0)	2 (50.0)	0 (0.0)	3 (75.0)	1 (25.0)	0 (0.0)	4 (100)	0 (0.0)	0 (0.0)
	S	17	13 (76.5)	3 (17.6)	1 (5.9)	14 (82.4)	2 (11.8)	1 (5.9)	16 (94.1)	0 (0.0)	1 (5.9)

D = Downstaging; C = Complete; U = Upstaging; S = Stable.

**Table 3:** Comparative analysis of gastrointestinal adverse events by treatment arm (TNT vs. CRT) with risk ratio estimates.

Adverse Event	Arm	Grade 0 *n* (%)	Grade 1 *n* (%)	Grade 2 *n* (%)	≥Grade 1 *n* (%)	RR (95% CI)	*p* Value (Fisher)
Nausea	TNT (*n* = 157)	112 (71.3)	35 (22.3)	10 (6.4)	45 (28.7)	3.15 (1.20–8.30)	0.012
	CRT (*n* = 44)	40 (90.9)	3 (6.8)	1 (2.3)	4 (9.1)	—	—
Vomiting	TNT (*n* = 157)	142 (90.4)	11 (7.0)	4 (2.5)	15 (9.6)	4.20 (0.57–30.95)	0.203
	CRT (*n* = 44)	43 (97.7)	1 (2.3)	0 (0.0)	1 (2.3)	—	—
Diarrhea	TNT (*n* = 157)	129 (82.2)	18 (11.5)	10 (6.4)	28 (17.8)	1.96 (0.73–5.29)	0.242
	CRT (*n* = 44)	40 (90.9)	2 (4.5)	2 (4.5)	4 (9.1)	—	—
ANY_GE1	TNT (*n* = 157)	—	—	—	52 (33.1)	2.08 (1.02–4.25)	0.038
	CRT (*n* = 44)	—	—	—	7 (15.9)	—	—

RR, risk ratio; CI, confidence interval. *p*-values were calculated using Fisher’s test.

**Table 4:** Univariate and trend analyses of clinical factors associated with grade ≥ 1 gastrointestinal adverse events.

Variable	Nausea ≥ Grade 1		Vomiting ≥ Grade 1		Diarrhea ≥ Grade 1	
	*p* (Global)	*p* (Trend)	*p* (Global)	*p* (Trend)	*p* (Global)	*p* (Trend)
Age	0.604	0.71	0.886	0.21	0.853	0.74
Sex	0.184	—	1.000	—	0.241	—
Tumor location	0.168	—	0.0054	—	0.9	—
Tumor grade	0.936	0.379	0.586	0.888	0.788	0.605
Initial stage	0.161	—	0.5478	—	0.1013	—
Postoperative stage	0.099	0.13	0.336	0.31	0.952	0.86
Stage modification	0.692	—	0.23	—	0.433	—

**Table 5:** Multivariable logistic and ordinal regression models for the composite endpoint (ANY_GE1).

Variable	Logistic Regression (ANY_GE1) OR (95% CI)	*p* Value	Ordinal Regression (Grade 0 → Grade 2) OR (95% CI)	*p* Value
Age (years)	1.01 (0.98–1.03)	0.50	1.01 (0.98–1.03)	0.47
Sex (F vs. M)	2.03 (1.05–3.77)	0.033	1.92 (1.03–3.59)	0.04
TNT (vs. CRT)	2.65 (1.08–6.53)	0.032	2.62 (1.04–6.60)	0.038
Upper tumor (vs. lower)	2.09 (0.91–4.88)	0.081	2.12 (0.93–4.86)	0.075
Middle tumor (vs. lower)	0.92 (0.42–2.01)	0.83	0.89 (0.41–1.95)	0.78

**Table 6:** Multivariable logistic models for gastrointestinal adverse events ≥ grade 1 (composite and individual endpoints).

Endpoint	Predictor	OR	95% CI	*p*-Value
ANY_GE1 (composite)	Age (per year)	1.007	0.977–1.037	0.6454
	Female (vs. Male)	1.913	1.021–3.584	0.043
	TNT (vs. CRT)	2.841	1.157–6.974	0.0227
Nausea ≥ grade 1	Age (per year)	1.009	0.978–1.042	0.5728
	Female (vs. Male)	1.718	0.882–3.349	0.1119
	TNT (vs. CRT)	4.373	1.449–13.202	0.0089
Vomiting ≥ grade 1	Age (per year)	0.978	0.932–1.025	0.3484
	Female (vs. Male)	1.036	0.364–2.954	0.9468
	TNT (vs. CRT)	4.051	0.511–32.085	0.1852
Diarrhea ≥ grade 1	Age (per year)	1.002	0.966–1.039	0.9334
	Female (vs. Male)	1.732	0.803–3.737	0.1614
	TNT (vs. CRT)	2.253	0.729–6.963	0.1581

**Table 7:** Sensitivity analyses of multivariable models for gastrointestinal adverse events.

Type of Analysis	Endpoint	Variable	Category	OR (If Applicable)	*p*-Value
A. Adverse events grade 2	Nausea grade 2	Sex	M/F	—	0.7628
		Tumor location	S/I/M	—	0.8418
		Tumor grade	G1–G3	—	0.8436
		Initial stage	IIA–IVA	—	0.3129
		Postoperative stage	I–pCR	—	0.989
		Stage modification	Down/Stable/Up/Complete	—	0.9706
	Vomiting grade 2	Sex	M/F	—	0.6392
		Tumor location	S/I/M	—	0.7569
		Tumor grade	G1–G3	—	0.5085
		Initial stage	IIA–IVA	—	0.255
		Postoperative stage	I–pCR	—	0.4284
		Stage modification	Down/Stable/Up/Complete	—	0.1842
	Diarrhea grade 2	Sex	M/F	—	0.7645
		Tumor location	S/I/M	—	0.4443
		Tumor grade	G1–G3	—	0.6213
		Initial stage	IIA–IVA	—	0.0143
		Postoperative stage	I–pCR	—	0.704
		Stage modification	Down/Stable/Up/Complete	—	0.3395
B. Multivariable logistic regression (AE ≥ grade 1)	AE ≥ grade 1	TNT vs. CRT	—	2.62	0.05
		Female vs. Male	—	1.87	0.073
		Tumor location (upper vs. lower)	—	2.25	0.09
		Age (per year)	—	1.01	0.73
C. Grade 2 TNT vs. CRT	Total grade 2	Treatment	TNT vs. CRT	2.72	0.256
D. Subgroup 50–80 years	AE ≥ grade 1	TNT vs. CRT	—	2.62	0.05
		Female vs. Male	—	1.87	0.073
		Tumor location (upper vs. lower)	—	2.25	0.09
		Age (per year)	—	1.01	0.73

## Data Availability

Data are contained within the article. All data are available by request to the corresponding authors.

## Abbreviations

The following abbreviations are used in this manuscript:

TNT	Total neoadjuvant therapy
CRT	Concurrent chemoradiotherapy
GI	Gastrointestinal
AEs	Adverse events
IMRT	Intensity-modulated radiotherapy
VMAT	Volumetric modulated arc therapy
CTCAE	Common terminology criteria for adverse events
IPTW	Inverse probability of treatment weighting
OR	Odds ratios
CI	Confidence intervals
CAPEOX	Capecitabine and oxaliplatin
FOLFOX	5-fluorouracil, oxaliplatin, and leucovorin
